# Leveraging machine learning for predicting acute graft-versus-host disease grades in allogeneic hematopoietic cell transplantation for T-cell prolymphocytic leukaemia

**DOI:** 10.1186/s12874-024-02237-y

**Published:** 2024-05-11

**Authors:** Gunjan Chandra, Junfeng Wang, Pekka Siirtola, Juha Röning

**Affiliations:** 1 Biomimetics and Intelligent Systems Group, University of Oulu, Pentti Kaiteran katu 1, 90570 Oulu, Finland; 2 Division of Pharmacoepidemiology and Clinical Pharmacology, Utrecht Institute for Pharmaceutical Sciences, Utrecht University, Utrecht, Netherlands

**Keywords:** Orphan diseases, Machine learning, Allogeneic hematopoietic cell transplantation, T-cell prolymphocytic leukemia, Acute graft-versus-host disease, Data size, Model performance

## Abstract

Orphan diseases, exemplified by T-cell prolymphocytic leukemia, present inherent challenges due to limited data availability and complexities in effective care. This study delves into harnessing the potential of machine learning to enhance care strategies for orphan diseases, specifically focusing on allogeneic hematopoietic cell transplantation (allo-HCT) in T-cell prolymphocytic leukemia. The investigation evaluates how varying numbers of variables impact model performance, considering the rarity of the disease. Utilizing data from the Center for International Blood and Marrow Transplant Research, the study scrutinizes outcomes following allo-HCT for T-cell prolymphocytic leukemia. Diverse machine learning models were developed to forecast acute graft-versus-host disease (aGvHD) occurrence and its distinct grades post-allo-HCT. Assessment of model performance relied on balanced accuracy, F1 score, and ROC AUC metrics. The findings highlight the Linear Discriminant Analysis (LDA) classifier achieving the highest testing balanced accuracy of 0.58 in predicting aGvHD. However, challenges arose in its performance during multi-class classification tasks. While affirming the potential of machine learning in enhancing care for orphan diseases, the study underscores the impact of limited data and disease rarity on model performance.

**Supplementary Information:**

The online version contains supplementary material available at 10.1186/s12874-024-02237-y.

## Introduction

T-cell prolymphocytic leukemia (T-PLL), constituting about 2% of mature lymphocytic leukemias in adults, exemplifies an orphan disease. These rare conditions, marked by their scarcity and a restricted patient population [2], present substantial challenges in research, diagnosis, and treatment [11]. The scarcity of data and resources for orphan diseases often hinders the development of effective care strategies. Hematopoietic stem cell transplantation (HSCT) is a commonly used therapeutic approach for treating various hematological disorders, including leukemia and lymphoma [6]. However, HSCT comes with a considerable risk of complications, and graft-versus-host disease (GvHD) is one of the most significant challenges faced by HSCT patients [10]. GvHD occurs when the donor’s immune cells recognize the recipient’s tissues as foreign and initiate an immune response against them [10]. The severity of GvHD can range from mild skin manifestations to life-threatening multiorgan dysfunction [10]. Therefore, accurate prediction of GvHD occurrence and severity is crucial for timely intervention and tailored treatment strategies [18].

In recent years, machine learning (ML) techniques have shown great promise in various healthcare domains, including disease prediction, diagnosis, and personalized treatment [7, 11, 14]. For instance, studies have demonstrated the effectiveness of ML models in predicting post-transplant complications and refining treatment approaches in hematopoietic cell transplantation [1, 18]. Additionally, ML has been explored for predicting acute GvHD, a common complication post allogeneic HCT and organ transplant [5, 18]. These studies have utilized various ML methods, such as decision trees, random forests, and neural networks, achieving significant advancements in patient care and treatment outcomes. However, despite these advancements, there remains a research gap in applying ML techniques to orphan diseases such as T-cell prolymphocytic leukemia [11]. While AI has shown promise in predicting and managing common diseases, limited research has been conducted in the context of orphan diseases.

This study aims to explore the potential of ML in improving orphan disease care, specifically focusing on allogeneic hematopoietic cell transplantation (allo-HCT) for T-cell prolymphocytic leukemia. By leveraging ML models, the study aims to enhance the prediction of acute GvHD grades following allo-HCT, which can aid in better patient management and treatment decisions [10, 18].

Acute GvHD can be classified into four grades based on clinical and histopathological criteria, commonly referred to as grades 1 to 4, as described by [8]. These grades represent: grade 1 (skin involvement), grade 2 (gastrointestinal tract involvement), grade 3 (liver involvement), and grade 4 (multiorgan involvement) [16]. Each grade presents unique challenges and requires tailored management strategies. Accurately predicting acute GvHD grades can aid in early intervention and guide personalized treatment approaches, ultimately improving patient outcomes. Several studies have investigated biomarkers and predictive models for acute GvHD [1, 12, 18]. In the present study, which is a part of the HTx project (EU Horizon 2020 funded project 2019-2024), we applied artificial intelligence as a tool to examine individualized predictions by searching complex relationships from high-dimensional data. The primary aim of HTx is to create a framework for the Next Generation Health Technology Assessment (HTA) to support patient-centered, societally oriented, real-time decision-making on access to and reimbursement for health technologies throughout Europe. To achieve the goals, we apply application of machine learning in this context to potentially advance orphan disease care and contribute to the understanding and treatment of rare conditions.

## Materials and methods

### Study design

This study was meticulously crafted to forecast the occurrence of aGvHD post-allo-HCT, focusing its predictive efforts on patients diagnosed with T-PLL.

The primary objective centered on developing robust predictive models tailored to anticipate and comprehend the onset of aGvHD in this specific cohort. By harnessing a nuanced understanding of this critical complication post-allo-HCT, the study aimed to contribute valuable insights into the prognosis and management of aGvHD in T-PLL patients.

Underpinning this endeavor was the utilization of advanced machine learning techniques, strategic curation of relevant features, and the adoption of a diverse range of classification algorithms. This methodological amalgamation aimed to not only forecast aGvHD onset but also delineate key contributing factors and patterns specific to T-PLL, fostering more informed clinical interventions and personalized patient care strategies.

#### Source of data

Data utilized in this study were obtained from the Center for International Blood and Marrow Transplant Research (CIBMTR) [4]. The dataset comprised clinical variables along with detailed information regarding acute GvHD grades [13].

#### Predictors

Initially, the raw dataset comprised 241 instances and encompassed 37 features. Supplementary Table S1 provides a comprehensive breakdown of the feature details. This dataset spanned data collected from 2008 to 2018. At the initial stage, a deliberate selection process excluded specific variables from the dataset. Variables were either identified as response variables or deemed irrelevant to the core research inquiry. Detailed information about the all variables and their status of inclusion is presented in Supplementary Table S1. This meticulous curation resulted in the identification of 11 informative features essential for baseline predictions.

#### Outcome

The main focus of this study was to predict the emergence of aGvHD (grades 2 to 4) within 100 days following allo-HCT, named ‘response_0to1_vs_2to4’, based on the 100 day marker ‘d100aGvHD24’. This condition, a notable complication post-transplant, presents considerable challenges in patient care and management. Predicting the timing and severity of aGvHD enables clinicians to anticipate and effectively manage potential complications, ultimately enhancing patient outcomes and their post-transplant quality of life.

In addition to predicting aGvHD occurrence (grades 2 to 4), two supplementary response variables, namely ‘response_0to2_vs_3to4’ and ‘response_0and1_vs_2_vs_3and4,’ were introduced in this study. These variables were carefully crafted based on 100-day marker variables, d100aGvHD24 and d100aGvHD34, with the explicit purpose of capturing the diverse patterns and varying grades of acute GvHD following allo-HCT.

The response variable, ‘response_0to2_vs_3to4’, was designed to discern and classify patients based on their likelihood of experiencing milder (grades 0 to 2) versus more severe (grades 3 to 4) acute GvHD. This distinction holds clinical significance as it aids in identifying patients at higher risk of developing severe complications post-transplantation, enabling tailored intervention strategies to mitigate potential adverse outcomes.

Similarly, the response variable, ‘response_0and1_vs_2_vs_3and4’, aimed to categorize patients into groups based on different combinations of acute GvHD grades (0, 1, 2, 3, or 4). This nuanced categorization allows for a more comprehensive understanding of the spectrum of acute GvHD severity and patterns, facilitating targeted therapeutic approaches and personalized patient care strategies.

By including these additional response variables, the study not only predicts the onset of aGvHD but also offers a more nuanced and granular assessment of the severity and patterns of this condition post-allo-HCT. This nuanced understanding is instrumental in tailoring patient care and interventions, thereby potentially improving clinical outcomes and patient well-being following transplantation.

#### Missing data and data splits

The dataset underwent further preprocessing, involving the removal of instances with missing responses, resulting in a refined dataset size of (216, 14) with 216 instances and 14 columns, consisting of 11 features and 3 response variables. To handle missing values within numeric features, mean imputations was adopted, wherein missing values were replaced with the respective means. Importantly, imputation was performed separately for the training and testing datasets to prevent any inadvertent data leakage. The division of data into training and testing subsets was accomplished through stratified k-fold cross-validation, employing a value of k set to 4. Where, in each iteration of 4-fold cross-validation:

- Each fold comprises approximately $216 / 4 = 54$ instances.
- 3 folds (approximately 162 instances) are used for training.
- 1 fold (approximately 54 instances) is used for testing.

Before training, only the training data was balanced using RandomOverSampler with a random state set to seed. The seed and code can be found in the supplementary document. This process ensures comprehensive and unbiased assessment of model performance across different subsets of the data.

### Statistical methods

#### Prediction models

The study embraced a diverse array of machine learning algorithms to comprehend and predict aGvHD following allo-HCT. The analysis and modeling were conducted using Python programming language. This included the utilization of three distinct models known for their efficacy in classification tasks from sklearn [15]:

- Linear Discriminant Analysis (LDA): LDA is a statistical technique emphasizing the linear combination of features to differentiate between classes, particularly efficient when classes are well-separated or normally distributed.
- k-Nearest Neighbors (KNN): KNN operates by classifying data points based on the majority class among their k-nearest neighbors in the feature space, making it a versatile and intuitive classification algorithm.
- Multilayer Perceptron (MLP): MLP, a type of artificial neural network, is adept at learning complex relationships within data by utilizing multiple layers of nodes, making it highly effective in nonlinear classification tasks.

The selection of these models was strategic, each offering distinct advantages in capturing different facets of the complex interactions influencing aGvHD prediction. By leveraging these varied algorithms, the study aimed to comprehensively explore and assess the predictive capabilities concerning acute GvHD post-allo-HCT. The machine learning models used in this study for predicting GvHD were implemented based on the code available in the GitHub repository [3].

#### Feature selection

Subsequently, feature selection techniques were applied to the subset of 11 features to enhance the model’s predictive performance and interpretability. The SelectKBest method from [15], which uses mutual information as the score function to assess statistical dependence between each feature and the target variable (in this case, the acute GvHD grade), was leveraged to identify the most informative features. This process allowed for the selection of the top *k* features with the highest mutual information scores, clearly indicating their relevance in predicting the target variable. Additionally, SelectKBest was employed to determine the optimal number of features that resulted in the best model performance for each classification task. The models were then ranked based on their performance, and the top three models are presented, along with the respective number of features used in each.

#### Performance metrics

For model evaluation, several performance metrics were employed, including training and testing balanced accuracy, testing F1 score, and testing Receiver Operating Characteristic Area Under the Curve (ROC AUC).

The F1 score was used to evaluate model performance in both binary and multiclass classification scenarios. In binary classifications such as ‘response_0to1_vs_2to4’ or ‘response_0to2_vs_3to4’, a weighted average F1 score was computed, considering class imbalances within the dataset. Meanwhile, in multiclass classification scenarios like ‘response_0and1_vs_2_vs_3and4’, a macro-average F1 score was utilized to weigh each class equally in the evaluation.

ROC AUC, on the other hand, quantified the model’s ability to distinguish between classes, providing crucial insights, especially in scenarios with multiple classes or imbalanced distributions. This metric assessed the models’ performance across different class predictions, complementing the F1 score evaluations.

These diverse metrics collectively offered insights into the models’ performance, accounting for various aspects such as class imbalances, model generalization, and class-wise distinctions, enabling a comprehensive evaluation of the model’s predictive capabilities.

In summary, the study utilized a robust methodology to analyze the outcomes of allo-HCT in patients with T-cell prolymphocytic leukemia. The dataset underwent preprocessing steps to address missing data, handle categorical variables, balance class distribution, standardize features, detect and remove outliers, and perform feature selection. Two new response variables were created to capture different acute GvHD grades, and only 11 relevant features were selected for baseline prediction. Multiple machine learning models were constructed and evaluated using various metrics, focusing on the selected informative features, to predict acute GvHD grades.

## Results

**Fig. 1:**
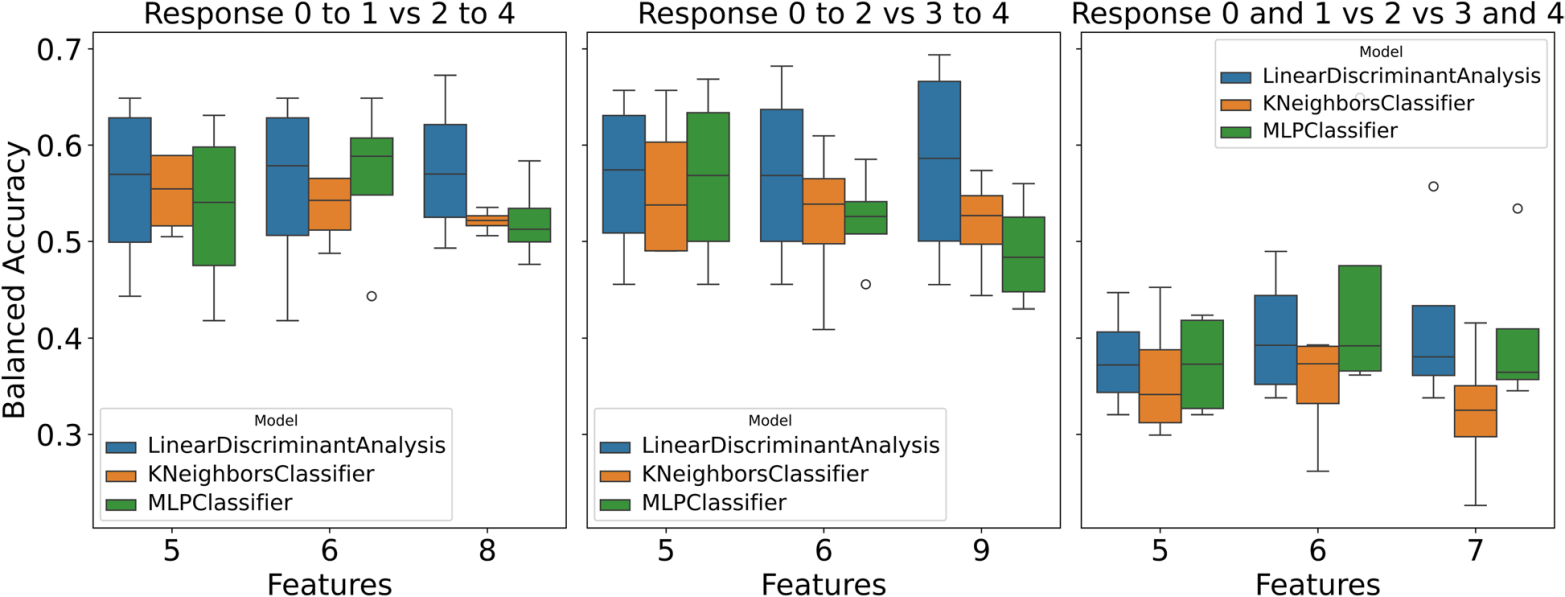
Performance of different machine learning models over different feature numbers for each response variable

This study presents the performance analysis of various models on three distinct response variables: ‘response_0to1_vs_2to4’ (class distribution: [0: 114, 1: 83]), ‘response_0to2_vs_3to4’ (class distribution: [0: 172, 1: 25]), and ‘response_0and1_vs_2_vs_3and4’ (class distribution: [0: 114, 1: 58, 2: 25]). Each model was subjected to training and testing using different numbers of features. The obtained results are depicted in Fig. 1 and Tables 1, 2, and 3, along with Supplementary Figures S1, S2, and S3 illustrating the performance of various ML models with significant features corresponding to different feature quantities.

**Table 1:** Model Performance Comparison with Varying Features for acute GvHD grades 0 to 1 vs 2 to 4

Model	Number of features	Training Balanced accuracy	Testing Balanced accuracy	Testing F1 score	Testing ROC AUC
KNeighborsClassifier	5	0.73	0.55	0.57	0.56
LinearDiscriminantAnalysis	5	0.58	0.56	0.57	0.59
MLPClassifier	5	0.59	0.53	0.55	0.59
KNeighborsClassifier	6	0.73	0.53	0.55	0.56
LinearDiscriminantAnalysis	6	0.59	0.56	0.57	0.58
MLPClassifier	6	0.58	0.57	0.58	0.58
KNeighborsClassifier	8	0.75	0.52	0.53	0.57
LinearDiscriminantAnalysis	8	0.61	0.58	0.59	0.59
MLPClassifier	8	0.91	0.52	0.53	0.55

**Table 2:** Model Performance Comparison with Varying Features for acute GvHD grades 0 to 2 vs 3 and 4

Model	Number of features	Training Balanced accuracy	Testing Balanced accuracy	Testing F1 score	Testing ROC AUC
KNeighborsClassifier	5	0.95	0.56	0.77	0.60
LinearDiscriminantAnalysis	5	069	0.57	0.75	0.60
MLPClassifier	5	0.67	0.57	0.75	0.59
KNeighborsClassifier	6	0.93	0.52	0.72	0.60
LinearDiscriminantAnalysis	6	0.70	0.57	0.73	0.60
MLPClassifier	6	0.85	0.52	0.76	0.55
KNeighborsClassifier	9	0.94	0.52	0.73	0.53
LinearDiscriminantAnalysis	9	0.71	0.58	0.74	0.58
MLPClassifier	9	1.00	0.49	0.78	0.50

**Table 3:** Model Performance Comparison with Varying Features for acute GvHD grades 0 and 1 vs 2 vs 3 and 4

Model	Number of features	Training Balanced accuracy	Testing Balanced accuracy	Testing F1 score	Testing ROC AUC
KNeighborsClassifier	5	0.51	0.36	0.35	0.50
LinearDiscriminantAnalysis	5	0.34	0.38	0.35	0.54
MLPClassifier	5	0.34	0.37	0.36	0.55
KNeighborsClassifier	6	0.52	0.35	0.34	0.49
LinearDiscriminantAnalysis	6	0.34	0.40	0.38	0.56
MLPClassifier	6	0.34	0.45	0.42	0.56
KNeighborsClassifier	7	0.52	0.32	0.31	0.52
LinearDiscriminantAnalysis	7	0.34	0.41	0.38	0.55
MLPClassifier	7	0.35	0.40	0.38	0.55

## Discussion

For the response variable, ‘response_0to1_vs_2to4’, three feature sets (Supplementary Figure S1) and models were evaluated, namely KNN, LDA, and MLP. The results are shown in Table 1. With a feature count of five, LDA achieved a balanced accuracy of 0.56, an F1 score of 0.57, and a ROC AUC of 0.59. Comparable performance metrics were observed for MLP and KNN.

When the feature count was increased to six, the models exhibited consistent performance for training, albeit with minor fluctuations in balanced accuracy, F1 score, and ROC AUC. However, MLP demonstrated a almost perfect balanced accuracy of 0.91 during training, suggesting potential overfitting as when the trained MLP model was tested using a test set, the best balanced accuracy it reached was 0.52 (see Table 1).

Similar patterns were observed for the response variable, ‘response_0to2_vs_3to4’; see Supplementary Figure S2 for selected variables and Table 2 for the results. LDA demonstrated a balanced accuracy of 0.69 during training with five feature values. This performance was sustained as the feature count increased to six and nine, with LDA maintaining robust performance across different feature counts. Moreover, MLP and KNN displayed comparable performance levels across various feature counts. Specifically, KNN and MLP demonstrating impressive balanced accuracy above 0.90 during training.

Regarding the response variable, ‘response_0and1_vs_2_vs_3and4’, the model’s performance noticeably diminished compared to the previous response variables; see Supplementary Figure S3 for selected variables and Table 3 for the results. All three models encountered challenges in attaining highly balanced accuracy, F1 score, and ROC AUC values. MLP demonstrated the highest performance among the models tested, achieving a balanced accuracy of 0.45, an F1 score of 0.42, and a ROC AUC of 0.56 with six features.

To summarize, selecting the response variable and the number of features substantially influence the model’s performance (Fig. 1). Generally, on average all models showcased superior performance. However, MLP exhibited signs of overfitting in certain instances showing that MLP could be too complex a model to be used with a small dataset. The findings underscore the criticality of feature selection and engineering in enhancing the predictive capabilities of the models.

While the model’s current performance might not be optimal, there’s room for improvement. Machine learning models possess the capacity to enhance their predictive capabilities, indicating their potential to directly assist in predicting acute GvHD. The ability to accurately identify the specific grade of acute GvHD following allo-HCT can have significant implications for treatment decisions and patient management. Different grades of acute GvHD may require tailored treatment approaches, such as immunosuppressive therapy or targeted interventions, to improve outcomes and reduce complications.

## Conclusion

In conclusion, this study highlights the potential of machine learning models in predicting acute GvHD grades following allo-HCT for T-PLL. The results demonstrate that machine learning algorithms, such as KNN, LDA, and MLP classifiers, can achieve varying degrees of accuracies ranging from 0.32 to 0.58 in predicting the occurrence of acute GvHD and its grades. These models, trained using carefully selected features, provide valuable tools for clinicians to make informed treatment decisions and improve patient management.

The rarity of T-cell prolymphocytic leukemia poses challenges in gathering sufficient data for analysis and prediction modelling. However, applying machine learning techniques provides a valuable tool for leveraging the available data and extracting meaningful insights. Using feature engineering techniques and various machine learning algorithms, researchers can uncover patterns and relationships within the data that may not be readily apparent through traditional statistical approaches. Moreover, it should be noted that simpler machine learning methods often perform as well with small datasets than complex models, as seen from this study.

The need for such tools becomes evident when considering the complexity and heterogeneity of acute GvHD. This condition can manifest differently and affect multiple organs, making accurate prediction and classification crucial for appropriate management. Machine learning models hold the capability to amalgamate an array of clinical, treatment, socio-economic predictors, alongside donor specifics and transplant intricacies, offering a comprehensive evaluation of acute GvHD’s risk and severity. This personalized approach can enhance treatment strategies, improve patient outcomes, and reduce the burden on healthcare resources.

However, it is crucial to acknowledge the limitations of this study, including the small dataset size, lack of holistic data, and the need for validation on larger cohorts. The rarity of T-cell prolymphocytic leukemia poses challenges in obtaining extensive data for training and testing the models. Collaboration among research institutions and the establishment of data-sharing initiatives can address these limitations and facilitate the development of more robust and accurate machine-learning models.

Additionally, the insights from the study on steroid-refractory intestinal aGvHD contribute to our understanding of complex immune-related conditions [9]. Steroid-refractory aGvHD remains a frequently fatal condition with limited knowledge about the mechanisms driving resistance to steroid treatments in the gut mucosa. The study’s analysis of gene expression profiles in rectosigmoid biopsies provides valuable molecular insights. The decreased expression of inhibitory genes (PDL1, IDO1, TIGIT) in steroid-refractory aGvHD indicates a disruption in immune regulation, likely contributing to the resistance to steroid treatment. This emphasizes the need for innovative approaches to tackle immune-related challenges [17]. Incorporating the insights from both studies, it becomes evident that a comprehensive understanding of immune regulation, stress responses, and environmental factors of both the patient and the donor is essential for developing more effective therapeutic strategies and improving patient outcomes in complex immune-related conditions such as aGvHD.

Nonetheless, this research sheds light on the potential of machine learning to improve orphan disease care. With continued efforts to collect and share data on rare diseases, the availability of more extensive and comprehensive datasets could enhance the performance of machine learning models in this domain. Collaborative initiatives and data-sharing platforms are crucial for overcoming the limitations posed by data scarcity in orphan disease research.

Overall, this study serves as a steppingstone in exploring the application of machine learning in orphan disease care. Further research and advancements in data collection, feature engineering, and model development are necessary to unlock the full potential of machine learning in improving outcomes for patients with orphan diseases like T-cell prolymphocytic leukemia.

### Supplementary Information

Supplementary Material 1.

## Data Availability

No datasets were generated or analysed during the current study.

## Code availability

Available at [3].

## References

[1] Arai Y, Kondo T, Fuse K, Shibasaki Y, Masuko M, Sugita J (2019). Using a machine learning algorithm to predict acute graft-versus-host disease following allogeneic transplantation. Blood Adv..

[2] Aronson J (2006). Rare diseases, orphan drugs, and orphan diseases. BMJ..

[3] Chandra G. ML_GvHD: Machine Learning Models for Predicting Graft-versus-Host Disease. https://github.com/gunjanchandra280395/ML_GvHD. Accessed 12 Apr 2024.

[4] CIBMTR. CIBMTR - Center for International Blood and Marrow Transplant Research. 2023. https://cibmtr.org. Accessed 26 Sept 2023.

[5] Cooper JP, Perkins JD, Warner PR, Shingina A, Biggins SW, Abkowitz JL (2022). Acute Graft-Versus-Host Disease After Orthotopic Liver Transplantation: Predicting This Rare Complication Using Machine Learning. Liver Transplant..

[6] Copelan EA (2006). Hematopoietic stem-cell transplantation. N Engl J Med..

[7] Esteva A, Kuprel B, Novoa RA, Ko J, Swetter SM, Blau HM (2017). Dermatologist-level classification of skin cancer with deep neural networks. Nature..

[8] Glucksberg H, Storb R, Fefer A, Buckner C, Neiman P, Clift R (1974). Clinical manifestations of graft-versus-host disease in human recipients of marrow from hl-a-matched sibling donor, s. Transplantation..

[9] Holtan SG, Shabaneh A, Betts BC, Rashidi A, MacMillan ML, Ustun C, et al. Stress responses, M2 macrophages, and a distinct microbial signature in fatal intestinal acute graft-versus-host disease. JCI Insight. 2019;4(17):e129762. 10.1172/jci.insight.129762 PMC6777917 31393854

[10] Jagasia M, Arora M, Flowers ME, Chao NJ, McCarthy PL, Cutler CS (2012). Risk factors for acute GVHD and survival after hematopoietic cell transplantation. Blood J Am Soc Hematol..

[11] Lee J, Liu C, Kim J, Chen Z, Sun Y, Rogers JR, et al. Deep learning for rare disease: A scoping review. J Biomed Inform. 2022;104227. 10.1016/j.jbi.2022.104227 36257483

[12] Levine JE, Logan BR, Wu J, Alousi AM, Bolaños-Meade J, Ferrara JL (2012). Acute graft-versus-host disease biomarkers measured during therapy can predict treatment outcomes: a Blood and Marrow Transplant Clinical Trials Network study. Blood J Am Soc Hematol..

[13] Murthy HS, Ahn KW, Estrada-Merly N, Alkhateeb HB, Bal S, Kharfan-Dabaja MA (2022). Outcomes of allogeneic hematopoietic cell transplantation in T cell prolymphocytic leukemia: a contemporary analysis from the center for international blood and marrow transplant research. Transplant Cell Ther..

[14] Obermeyer Z, Emanuel EJ (2016). Predicting the future-big data, machine learning, and clinical medicine. N Engl J Med..

[15] Pedregosa F, Varoquaux G, Gramfort A, Michel V, Thirion B, Grisel O (2011). Scikit-learn: Machine Learning in Python. J Mach Learn Res..

[16] Pidala J, Vogelsang G, Martin P, Chai X, Storer B, Pavletic S (2012). Overlap subtype of chronic graft-versus-host disease is associated with an adverse prognosis, functional impairment, and inferior patient-reported outcomes: a Chronic Graft-versus-Host Disease Consortium study. Haematologica..

[17] Scarola SJ, Perdomo Trejo JR, Granger ME, Gerecke KM, Bardi M (2019). Immunomodulatory effects of stress and environmental enrichment in Long-Evans rats (Rattus norvegicus). Comp Med..

[18] Tang S, Chappell GT, Mazzoli A, Tewari M, Choi SW, Wiens J (2020). Predicting acute graft-versus-host disease using machine learning and longitudinal vital sign data from electronic health records. JCO Clin Cancer Inform..

