# Supplementary material for: Leveraging machine learning for predicting acute graft-versus-host disease grades in allogeneic hematopoietic cell transplantation for T-cell prolymphocytic leukaemia
Source: BMC Med Res Methodol. 2024 May 11;24:112. doi: 10.1186/s12874-024-02237-y (PMC11088760; doi:10.1186/s12874-024-02237-y)
Supplement: Supplementary file 1 — Supplementary Material 1. [file 12874_2024_2237_MOESM1_ESM.pdf]

Supplementary Material

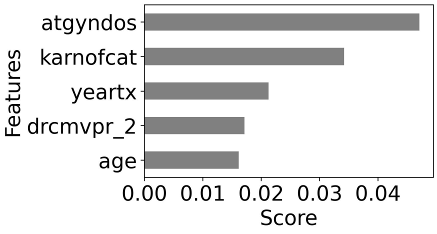

(a)

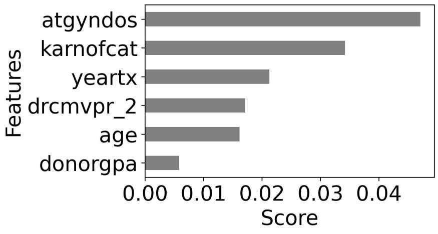

(b)

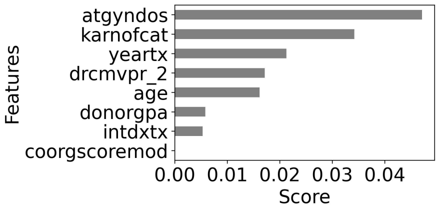

(c)

Fig. S1: Feature importance plots for response 0 to 1 vs 2 to 4 with 5, 6, and 8 number of features, respectively.

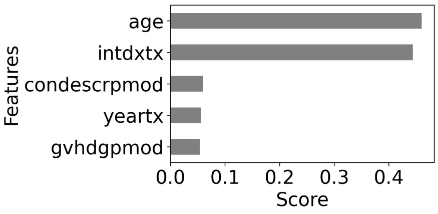

(a)

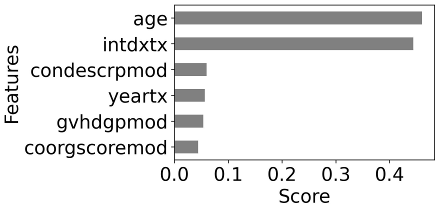

(b)

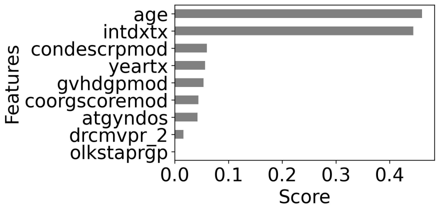

(c)

Fig. S2: Feature importance plot for acute GvHD grades 0 to 2 vs 3 and 4 with 5, 6, and 9 number of features, respectively.

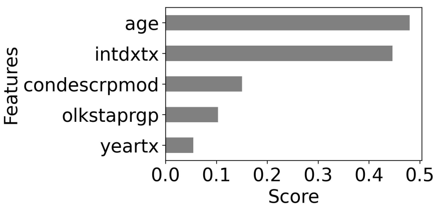

(a)

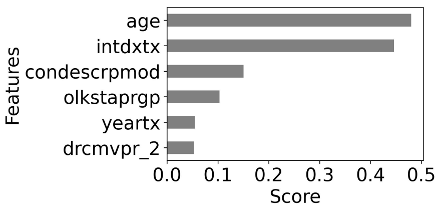

(b)

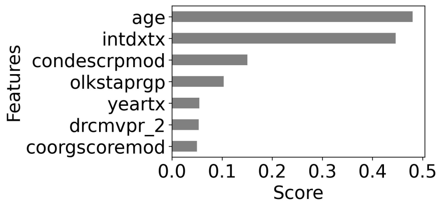

(c)

Fig. S3: Feature importance plot for acute GvHD grades 0 and 1 vs 2 vs 3 and 4 with 5, 6, and 7 number of features, respectively.

Table S1: Data description

Variable	Description	Value	Label	Used in this study
yeartx	Year of transplant	Continuous		Yes
age	Age, median (range), yrs	Continuous		Yes
intxsurv	Time from HCT to death/last follow-up date	Continuous		No
GvHDgpmod	GvHD prophylaxis	6	CNI + MMF +/- others (except post Cy)	Yes
		7	CNI + MTX +/- others (except MMF, post Cy)	
		8	CNI + others (except MMF, MTX, post Cy)	

		10<br>88	Post Cy +/- others prophylaxis<br>Other prophylaxis	
karnofcat	Karnofsky score	1<br>2<br>99	90-100<br><90<br>Missing	Yes
intdxtx	Time from diagnosis to HCT	Continuous		Yes
donorgpa	Donor type	1<br>2<br>3<br>4<br>9<br>98	HLA id-sib<br>Haplo<br>URD 8/8<br>URD 7/8<br>Cord blood<br>Other related	Yes
atgyndos	ATG/Campath use	1<br>2<br>99	Yes<br>No<br>Missing	Yes
dead	Overall survival	0<br>1<br>99	No<br>Yes<br>Missing	No
rel	Relapse	0<br>1<br>99	No<br>Yes<br>Missing	No
trm	Treatment related mortality	0<br>1<br>99	No<br>Yes<br>Missing	No
dfs	Disease free survival	0<br>1<br>99	No<br>Yes<br>Missing	No
aGvHD	Acute 2-4 GvHD	0<br>1<br>99	No<br>Yes<br>Missing	No
cGvHD	Chronic GvHD	0<br>1<br>99	No<br>Yes<br>Missing	No
intxrel	Time from HCT to relapse, months	Continuous		No
intxgf	Time between transplant and graft failure, months	Continuous		No
drcmvpr_2	Donor/recipient CMV serostatus	0<br>1	+/+<br>+/-	Yes

		2<br>3<br>9<br>97<br>99	-/+<br>-/-<br>Cord blood<br>Not available before 2007<br>Missing	
condescrpmod	Conditioning regimen intensity	1<br>2<br>3<br>4<br>99	MAC-TBI<br>MAC-Chemo<br>RIC/NMA-TBI<br>RIC/NMA-Chemo<br>Missing	Yes
olkstaprgp	Disease status at HCT	2<br>4<br>5	Complete Remission (CR)<br>(PR/(nPR)<br>No response/stable (NR/SD)/Progression	Yes
ageres	Age, years	Categorical	<60<br>60+	No
intdxtx6gp	Time from diagnosis to transplant, months	Categorical	<6 months<br>6-11 months<br>>= 12 months	No
coorgscoremod	HCT-CI	0<br>1<br>2<br>3<br>4<br>5<br>99	0<br>1<br>2<br>3<br>4<br>5<br>Missing	Yes
intxaGvHD	Time between transplant and acute GvHD, months	Continuous		No
gf	Primary graft Failure	0<br>1<br>99	No<br>Yes<br>Missing	No
dwoaGvHD	Competing risk: aGvHD	0<br>1<br>99	No<br>Yes<br>Missing	No
dwocGvHD	Competing risk: cGvHD	0<br>1<br>99	No<br>Yes<br>Missing	No

dwogf	Competing risk: Graft Failure	0<br>1<br>99	No<br>Yes<br>Missing	No
agegpan	Age Groups	2<br>3<br>4<br>5<br>6<br>7	18-29<br>30-39<br>40-49<br>50-59<br>60-69<br>70+	No
dccn	Dummy CCN	Continuous		No
dcrid	Dummy Crid	Continuous		No
intxaGvHD34	Time between transplant and acute GvHD 3-4, months	Continuous		No
aGvHD34	Acute 3-4 GvHD	0<br>1<br>99	No<br>Yes<br>Missing	No
dwoaGvHD34	Competing risk: aGvHD 3-4	0<br>1<br>99	No<br>Yes<br>Missing	No
d100aGvHD24	Day 100 indicator of Acute 2-4 GvHD (MVA)	0<br>1<br>99	No<br>Yes<br>Missing	Response variable
d100aGvHD34	Day 100 indicator of Acute 3-4 GvHD (MVA)	0<br>1<br>99	No<br>Yes<br>Missing	Response variable
grpcod	Cause of death	0<br>1<br>2<br>3<br>4<br>5<br>6<br>7<br>8<br>9<br>10	Alive<br>Primary Disease<br>Graft Failure<br>GvHD<br>Infection<br>Ipn/ARDS<br>Organ Failure<br>Organ Toxicity<br>Secondary Malignancy<br>Other Cause<br>Unknown	No
condintmod	Conditioning regimen intensity	1<br>2	MAC<br>RIC	No
